# Embedding dual function into molecular motors through collective motion

**DOI:** 10.1038/srep44288

**Published:** 2017-03-10

**Authors:** Nen Saito, Kunihiko Kaneko

**Affiliations:** 1 Universal Biology Institute, Graduate School of Science, The University of Tokyo, 7-3-1 Hongo, Bunkyo-ku, Tokyo, 113-0033, Japan; 2 Research Center for Complex Systems Biology, Graduate School of Arts and Sciences, The University of Tokyo, 3-8-1 Komaba, Meguro-ku, Tokyo, 153-8902, Japan

## Abstract

Protein motors, such as kinesins and dyneins, bind to a microtubule and travel along it in a specific direction. Previously, it was thought that the directionality for a given motor was constant in the absence of an external force. However, the directionality of the kinesin-5 Cin8 was recently found to change as the number of motors that bind to the same microtubule is increased. Here, we introduce a simple mechanical model of a microtubule-sliding assay in which multiple motors interact with the filament. We show that, due to the collective phenomenon, the directionality of the motor changes (e.g., from minus- to plus- end directionality), depending on the number of motors. This is induced by a large diffusive component in the directional walk and by the subsequent frustrated motor configuration, in which multiple motors pull the filament in opposite directions, similar to a game of tug-of-war. A possible role of the dual-directional motors for the mitotic spindle formation is also discussed. Our framework provides a general mechanism to embed two conflicting tasks into a single molecular machine, which works context-dependently.

Microtubule-based motors such as kinesins and dyneins are essential to a variety of processes, e.g., molecular transportation and mitotic spindle formation. The force generation is mediated by the walk of a unidirectional motor toward the plus/minus end of the microtubule (MT), following adenosine triphosphate (ATP) hydrolysis. Intuitively, highly processive and non-diffusive motor walks may be advantageous for efficient force generation. However, motors exhibiting a biased random walk toward a specific end with a large diffusion coefficient have also been reported^1^,^2^,^3^,^4^. The questions therefore arise as to why motors with such biased random walks exist in nature and whether or not such motors play a specific role.

It was long believed that a given type of motor protein travels in a specific direction along an MT, and that this directionality remains constant in the absence of an external force. Quite recently, however, kinesin-5 Cin8, which is a tetrameric motor protein purified from budding yeast that can cross-link to two MTs, was found to exhibit directionality switching, depending on the number of motors bound to the same MT^1^. Based on the other kinesin-5 family members, Cin8 was expected to exhibit plus-end directionality, leading to outward-force generation, which separates anti-parallel MTs with their minus-end leading during mitotic spindle formation^5^. However, Roostalu *et al*.^1^ showed that a single Cin8 molecule exhibits a biased random walk with a large diffusion constant toward the minus end. When multiple Cin8’s function as a team by cross-linking antiparallel MTs simultaneously, they transport anti-parallel MTs so that each MT slides with its minus end leading, which represents the expected plus-end directionality of Cin8. These researchers also conducted an MT sliding assay, where multiple surface-immobilized motors collectively bind to MTs and transport them, and demonstrated that the motors exhibit minus-end directionality for a small number of binding motors, while they exhibit plus-end directionality for a large number of binding motors. After this finding, similar directionality switch depending on the number of motors has been also reported for kinesin-14 KlpA^6^ and kinesin-5 Cut7^7^, which suggests that motor with such “dual-directionality” (i.e., ability to show both directionality depending on conditions) is general and plays some important roles. Note that the dual directionality exhibited by Cin8 differs from bidirectionality^8^,^9^, where both plus- and minus-end directed motions can coexist under the same conditions.

Similarly, the emergence of a novel motility mode, other than the directionality transition, induced by team formation has also been reported in several experimental studies^10^,^11^,^12^. In those cases, despite weakly biased or even non-biased diffusion in the walk of an individual motor, a team of motors exhibits a highly directional movement. However, the physical principle underlying both the directionality transition and the emergence of the directionality has not yet been uncovered.

In this Paper, we introduce a mathematical model for a motor-filament system and demonstrate that directional collective motion contrary to the built-in directionality of a single motor can emerge in the absence of any external forces. This directionality transition emerges under a large diffusional component in the directional walk and asymmetry in the intra-molecular strain-dependent detachment. In addition, the collective directional motion of motors with non-biased diffusion is explained. The proposed model provides a representative example of a mechanism for embedding dual tasks into a single molecular machine, which will elucidate the role of a large diffusive component in the walk of a processive motor. The emergence of the collective motion demonstrated here is independent of, and fundamentally different from, that induced by the entropic force produced by a diffusible cross-linker^13^ or the jamming effect that facilitates opposite collective directional motion^14^, since neither of those effects occur in the MT sliding assay considered in this study.

## Model

**Figure 1:**
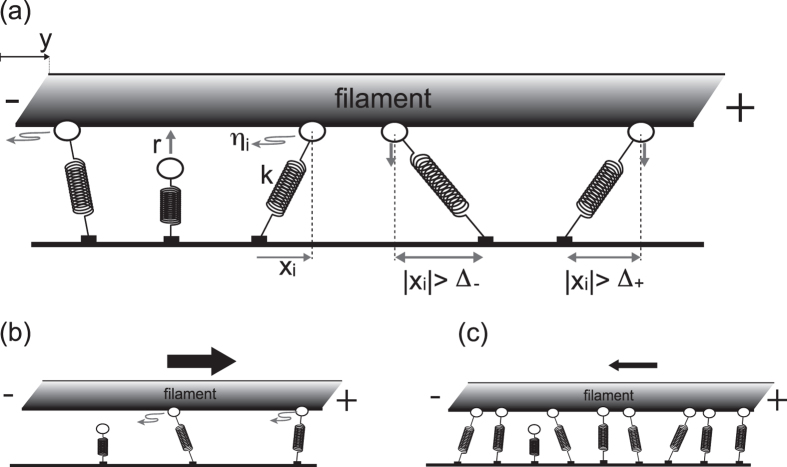
Stochastic model for microtubule sliding assay. (**a**) Schematic representation of the model. We address if and how (**b**) the minus-end oriented directionality of the motor switches to (**c**) plus-end directionality as the number of motors *N* increases. Black arrows above the filament indicate the direction of the movement of the filament.

Because our study is motivated by the MT sliding assay reported in Roostalu *et al*.^1^, we consider a situation in which a polar filament with plus and minus ends slides along a glass surface on which multiple motor molecules are tethered, as shown in Fig. 1. A finite number of motors can interact simultaneously with the filament. For simplicity, and to illustrate the basic mechanism for the directionality transition, we consider a one-dimensional situation, where *N* identical motors *X*_1_, *X*_2_, …, *X*_*N*_ can be attached to a single rigid rod. As shown in Fig. 1(a), each cross-linking motor molecule is represented by a spring tethered to a bottom surface and a motor, which can bind to the MT and walk along it. Therefore, the motor not only produces a force-generating stroke, but also moves via a random walk along the filament, which is the major difference between this model and previous models of motor-filament systems^9^,^15^,^16^,^17^,^18^. Under such a situation, we consider if and how the motor’s directionality switches as *N* increases, as illustrated in Fig. 1(b,c).

The displacement along the filament between the bound motor and the tethering point (i.e., the extension of the spring along the filament) is denoted by *x*_*i*_ (*i* = 1, …, *N*) for the *i*th motor, whereas the filament coordinate is denoted by *y*, as illustrated in Fig. 1(a). For an unbound motor, *x*_*i*_ = 0 is assigned. The filament then slides along the surface, pulled by multiple bound motors with force 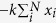, where *k* is a spring constant. The time evolution for the filament with the friction coefficient *γ*_*y*_ under over-damped conditions is given by


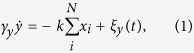


where *ξ*_*y*_(*t*) is the thermal random force applied to the filament as 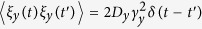. Each motor bound to the filament moves via a one-dimensional random walk along the filament, the driving force of which is generated by binding of the leading head to the MT associated with the motor step. The driving force is assumed to arise from thermal activation or ATP hydrolysis, and is denoted by *η*_*i*_ = *η(x*_*i*_, *t*). For motors bound to the filament, it is natural to assume a no-slip condition: the relative distance between each bound motor *x*_*i*_ and the filament *y* does not change unless *η*_*i*_ causes the *i*-th motor’s “walk” on the filament.


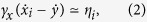


where *γ*_*x*_ is the friction coefficient of the motor with the surrounding medium, which is considered to be much smaller than that of filament *γ*_*y*_. This indicates that the relative distance does not change if *η*_*i*_ = 0. From Eqs 1 and 2, the time evolution for the filament and the bound motor is given by


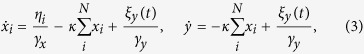


where *κ* = *k/γ*_*y*_. The driving force of the walk *η*_*i*_ is modeled as 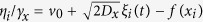. Here, *v*_0_ represents the built-in walk velocity, 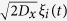 the diffusive mode in the motor’s walk with diffusion constant *D*_*x*_, and 〈*ξ*_*i*_(*t)ξ*_*j*_(*t*′)〉 = *δ*_*i,j*_*δ(t* − *t*′). In addition, *f(x*_*i*_) represents how the motor’s walk is biased depending on the intra-molecular strain *x*_*i*_. For example, the motor with *x*_*i*_ > 0, which is pulled from the minus-end due to the intra-molecular strain, is more likely to proceed toward the minus-end in the next step of the motor’s walk than the motor with *x*_*i*_ < 0 does. Hereafter, we mainly take *f(x*) as a linear function: *f(x*) = *a*_0_*x*. The force-velocity relation of the single motor has been measured and discussed in several experimental papers^19^,^20^. The corresponding relationship in our model is discussed in Supplementary Information. A more precise derivation of the model is also given in Supplementary Information.

The bound motor can be detached from the filament in either a force-dependent or a force-independent manner. For the force-dependent detachment, we assume that the motor becomes detached if *x* > Δ_+_ or *x* < −Δ_−_, with given positive parameters Δ_+_ and Δ_−_, whereas for the force-independent detachment, every bound motor is detached at the rate 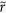, independent of *x*_*i*_. In Supplementary Information, we have also examined the exponential dependency of the detachment rate on the force, to confirm that the results below are not altered by the choice of the detachment rule. On the other hand, an unbound motor can be attached to the filament at *x* = 0 with rate *r*.

## Results

### The directionality transition

**Figure 2:**
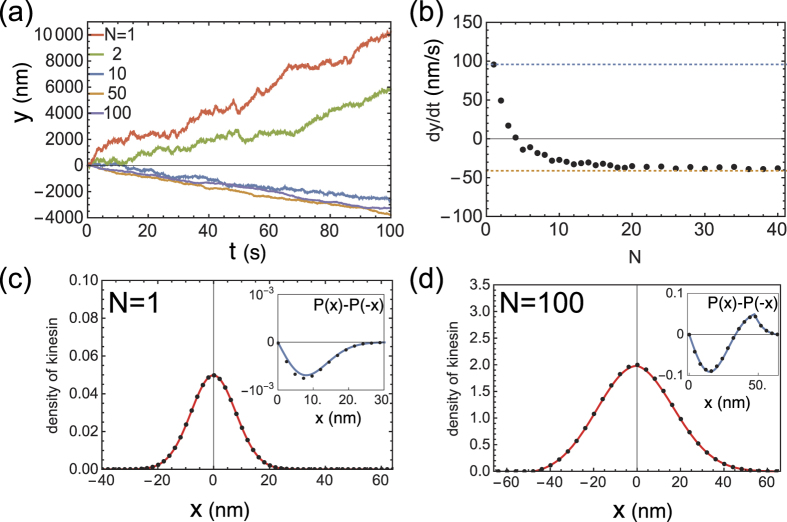
Behavior of model with asymmetric strain-dependent detachment. (**a**) Time series of filament coordinate *y* for varying *N*. (**b**) 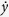 dependency on *N*. Both (**a**,**b**) exhibit directionality transitions with increasing *N*. The upper and bottom dashed lines in (**b**) indicate theoretical estimates for *N* = 1 and for a sufficiently large *N*, respectively. (**c**,**d**) show the *x* distribution *P(x*) for *N* = 1 and 100, respectively. The lines and dots represent theoretical estimates and simulation results, respectively. In the inset, the difference in *P(x*) for *x* > 0 and *x* < 0, i.e., *P(x*) − *P*(−*x*), is plotted to make the asymmetry in *P(x*) clearer. The following parameters are used: *v*_0_ = −100 [nm/s]; *D*_*x*_ = 3 × 10^4^ [nm^2^/s]; *D*_*y*_ = 4 × 10^4^ [nm^2^/s]; *κ* = 1000 [s^−1^] (*k* = 0.1 [pN/nm] and *γ*_*y*_ = 10^−4^ [pN · s/nm]); Δ_+_ = 64 [nm]; Δ_−_ = 48 [nm]; *r* = 20 [*s*^−1^]; 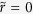; and *a*_0_ = 90 [s^−1^].

Without loss of generality, we consider the case *v*_0_ < 0, which indicates that each individual motor takes a biased random walk toward a minus-end, whereas the case *v*_0_ = 0 will be discussed later. Because of the walking direction asymmetry of each motor, the filament always moves toward the plus-end and a directionality transition is impossible unless some asymmetry is introduced. Indeed, a directionality transition is observed for increased *N*, under the presence of: (i) an asymmetric strain-dependent detachment Δ_+_ > Δ_−_ or (ii) an asymmetric dependency of the motor’s velocity upon the intramolecular strain |*f(x*)| > |*f*(−*x*)|. Here, we primarily address case (i). Figure 2(a,b) show the time series of *y* for various *N* and the *N* − 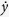 relation from the results of numerical simulations of Eq. 3, respectively, and indicate that the sign of 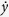 changes as *N* increases (see also Video S1 in the Supplementary Information). Interestingly, this directionality transition is not observed for *D*_*x*_~0, which suggests that diffusion in the motor’s walk induces the transition, because of a cooperative phenomenon.

Next, we solve the proposed model analytically to elucidate the mechanism of the directionality transition. Steady state solutions of the model are obtained for both *N* = 1 and infinite *N*.

For simplicity, we consider only 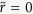 here, although we also have confirmed the transition in the directionality for 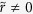 (data not shown). For *N* = 1, the time evolution of the motor distribution *P(x, t*) is given by the Fokker-Planck equation (equivalent to Equation 3), such that


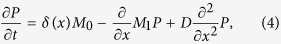


where *D* = *D*_*x*_ + *D*_*y*_, *M*_0_ = *r(N* − *N*_*b*_), 
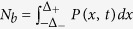
, *M*_1_ = *v*_0_ − *f(x*) − *κx*, and *δ(x*) is the Dirac delta function. The stationary distribution of the above equation is given by *P(x*) = *M*_0_*ρ(x*), where


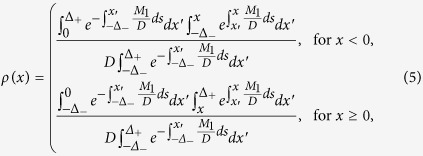


and 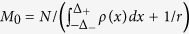. The explicit form of the distribution is given in the Supplementary Information. The analytical estimates for *N* = 1 agree rather well with the results of the stochastic simulations, as shown in Fig. 2(c).

For finite *N*, the time evolution of *P(x, t*) is given by an *N*-dimensional differential equation, which is very difficult to solve. As *N* goes to infinity, however, the time evolution of *P(x, t*) is reduced to a one-body problem, which is governed by the same equation as Eq. (4), with *D* = *D*_*x*_ (see Supplementary Information) and *M*_1_ = *v*_0_ − *f(x*) − *κ*〈*x*〉. Here 〈*x*〉 is defined by 
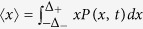
 and *M*_1_ contains the term 〈*x*〉, which cannot be determined without knowledge of *P(x*); thus, Eq. 4 becomes a nonlinear Fokker-Planck equation. By solving the self-consistent equation 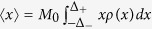, where *ρ(x*) on the right hand side is given in Eq. 5, we obtain 〈*x*〉 and *ρ(x*). The estimated analytical result agrees rather well with that of the stochastic simulations for sufficiently large *N*, as shown in Fig. 2(d).

These analytical results illustrate the directionality transition mechanism. For *N* = 1, the *x*_*i*_ distribution has a significantly sharper profile than that for *N* = 100, which indicates that the intra-molecular strain due to the extension of the motor is immediately released. That is, the filament can move immediately in the direction in which it is pulled by the motor, and *x* always remains close to zero. Thus, the asymmetry in Δ_+_/Δ_−_ cannot manifest. The directionality is, therefore, determined according to the built-in walk direction, which is consistent with the slightly biased distribution in Fig. 2(c) with the negative mean 〈*x*〉: although the distribution seems to be almost symmetric, the non-zero value of *P(x*) − *P*(−*x*), as is shown in the inset of Fig. 2(c), indicates that the distribution is slightly skewed. Accordingly, 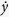 is positive. On the other hand, for a sufficiently large *N* and large *D*_*x*_, the distribution of *x*_*i*_ is significantly broader, which indicates that the multiple motors pull the filament in opposing directions, similar to a game of tug-of-war. Thus, the intra-molecular strain in each motor is released only a little. This “frustrated” motor configuration causes the asymmetry in Δ_+_/Δ_−_ to manifest. Specifically, in the case of Δ_+_ > Δ_−_, the backward force with respect to the walking direction (i.e., the plus-end-oriented force) applied to the motors with *x* < 0 causes them to detach more easily than the forward force applied to the motors with *x* > 0. This asymmetry allows for a slightly longer tail at *x* > 0 compared to that at *x* < 0 in the distribution of Fig. 2(d). Therefore, the motor distributions are positively biased at the tails, so the mean 〈*x*〉 is positive and 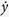 is negative, as illustrated in the insets of Fig. 2(d).

**Figure 3:**
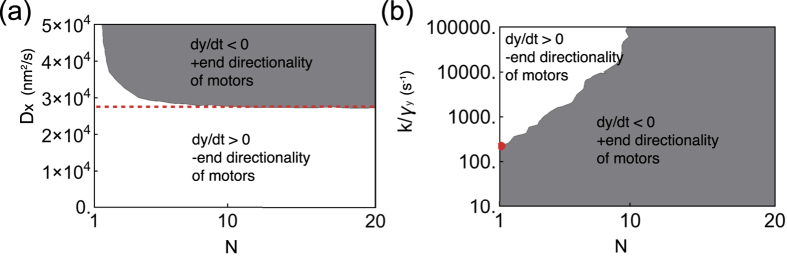
Directionality transition phase diagrams. The white and shaded regions represent positive and negative 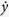 (i.e., minus- and plus-end-oriented directionality of the motor), respectively. In (**a**), the dashed line indicates the theoretical estimate of the critical value of *D*_*x*_, below which directional transition never emerges. The red dot in (**b**) indicates the theoretically estimated critical point for *N* = 1. The parameters are the same as those for Fig. 2.

Figure 3(a,b) show phase diagrams of the regime for plus- or minus-end-directed filament movement, which were obtained numerically. As shown in Fig. 3(a), large diffusivity (i.e., large *D*_*x*_) in the walk, rather than a random thermal force *ξ*_*y*_, is crucial for the emergence of the directionality transition. This is because large *D*_*x*_ leads to a broader *x* distribution, which reflects a highly frustrated “tug-of-war” situation of the motors. If *D*_*x*_ is small, the motors move with the built-in direction concurrently, yielding a sharper *x* distribution and smaller intra-molecular strains. Thus, the directionality transition is absent. This is also true even in the presence of a thermal force applied to the filament (i.e., *ξ*_*y*_) much larger than *v*_0_, as this force does not change the relative distances between the motors.

In addition to the directionality transition with increased *N* discussed above, the phase diagram in Fig. 3(b) predicts another directionality transition with a decrease in *κ* = *k/γ*_*y*_ (e.g., an increase in *γ*_*y*_ for fixed *k*). We have also confirmed this transition in the analytical calculation for *N* = 1, as shown by the red dot in Fig. 3(b). This transition can be explained as follows. Low *κ* signifies low filament mobility and, thus, the strain in the stretching motor is not released by the filament movement, leading to a broader *x* distribution. As a result, the effect of the asymmetry in the strain-dependent detachment Δ_+_/Δ_−_ becomes prominent, which allows for a slightly longer tail at *x* > 0 in the distribution; thus, the contribution of the tail to the mean 〈*x*〉 surpasses that of *v*_0_.

In addition, we can also consider an extreme low-mobility situation, where the filament is fully immobilized through application of an external force *F(x*), which results in 
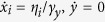
 and 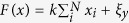. Surprisingly, even for *N* = 1, this *F(x*) is positive on average. That is, 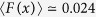 [pN] for the parameters employed for Fig. 2, which indicates that, on average, a positive *F(x*) is required to immobilize the filament. This is despite the fact that the filament velocity is positive in the absence of *F(x*). This counterintuitive result can also be understood by noting that the increase in the strain (i.e., the tails of *P(x*)) through immobilization of the filament causes the asymmetry in Δ_+_/Δ_−_ to manifest.

Even without the asymmetry in the detachment process, directionality transitions can also be observed with increased *N* by introducing case (ii), the asymmetric dependency of the motor’s velocity upon the intramolecular strain |*f(x*)| > |*f*(−*x*)|, i.e., *f(x*) = *a*_+_*x* (if *x* ≥ 0) and *a*_−_*x* (if *x* < 0), with *a*_+_ < *a*_−_. In this case, the same directionality transition as in case (i) Δ_+_ > Δ_−_ is obtained via the analytical estimates, as shown in Figure S1 in the Supplementary Information.

### Emergence of directionality

**Figure 4:**
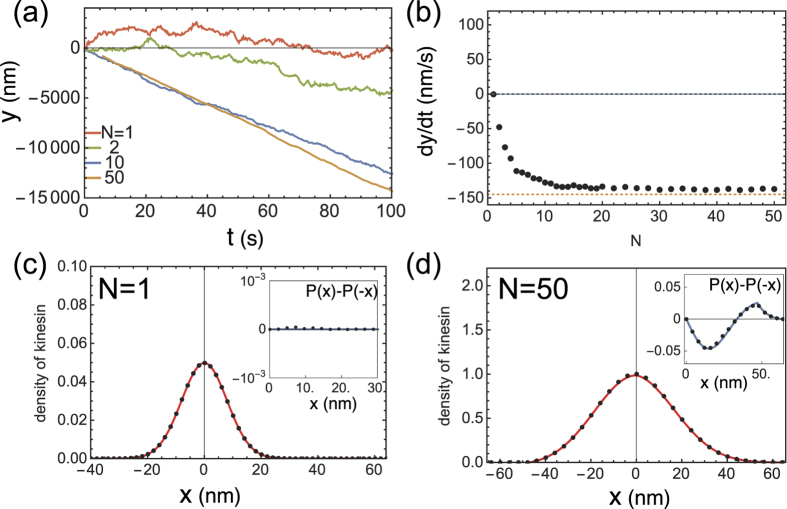
Behavior of model with asymmetric strain-dependent detachment for *v*_0_ = 0 [nm/s]. (**a**) Time series of filament coordinate *y* for varying *N*. (**b**) 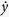 dependency on *N*. Both (**a**,**b**) illustrate the directionality transition with increasing *N*. The upper and bottom dashed lines in (**b**) indicate theoretical estimates for *N* = 1 and for sufficiently large *N*, respectively. (**c**,**d**) show the *x* distribution *P(x*) for *N* = 1 and 50, respectively. The lines and dots represent theoretical estimates and simulation results, respectively. In the inset, the difference in *P(x*) for *x* > 0 and *x* < 0, i.e., *P(x*) − *P*(−*x*), is plotted to make the asymmetry in *P(x*) clearer The following parameters are used: *v*_0_ = 0 [nm/s]; *D*_*x*_ = 3 × 10^4^ [nm^2^/s]; *D*_*y*_ = 4 × 10^4^ [nm^2^/s]; *κ* = 1000 [s^−1^] (*k* = 0.1 [pN/nm] and *γ*_*y*_ = 10^−4^ [pN · s/nm]); Δ_+_ = 64 [nm]; Δ_−_ = 48 [nm]; *r* = 20 [*s*^−1^]; 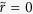; and *a*_0_ = 90 [s^−1^].

Finally, by setting *v*_0_ = 0, we confirm that the filament moves via non-biased diffusion for *N* = 1, whereas collective directional motion of the filament toward the minus-end emerges for large *N*, as shown in Fig. 4(a,b). In this case, the distribution *P(x*) is symmetric for *N* = 1 as illustrated by the inset in Fig. 4(c), whereas the asymmetry in *P(x*) manifests for *N* = 50 in Fig. 4(d) and its inset. This would explain the emergence of collective directionality reported in several experimental studies^10^,^11^,^12^.

## Discussion

In this Paper, we have investigated a mathematical model of a motor-filament system and shown that, with a large diffusivity in the motor’s walk and asymmetry in the strain-dependent detachment (Δ_+_/Δ_−_) or in the force-velocity relation (*a*_+_/*a*_−_), the directionality in the filament movement can be reversed via an increase in the number of motors *N*. Although neither of these asymmetries has yet been measured for Cin8, asymmetry in the force-dependent detachment or the force-velocity relation have been reported for several motor proteins^19^,^20^,^21^,^22^,^23^,^24^,^25^, which suggests that the assumption of asymmetry is not unnatural. Under such an asymmetry and for small *N*, the intra-molecular strains in the motors are immediately released by the filament movement; thus, the motors walk in the built-in walk direction, i.e., towards the minus-end. On the other hand, when *N* increases, the diffusive nature of the processive motors leads to a frustrated motor configuration in which multiple motors pull the filament in opposite directions; thus, the intra-molecular strains in each motor are increased. These inter- and intra-molecular frustrations amplify the asymmetry in the strain-dependent properties of the motors and generate the opposite motility mode. Thus, the motors act as plus-end-directed motors, although their average movement is toward the minus-end. Similar to the several macroscopic models of motor-filament system^26^,^27^,^28^,^29^, in the model presented in this study, detailed molecular processes of ATP consumption are coarse grained; however, we believe that it captures the essence of the changes in the motility mode as a cooperative phenomenon in the motor-filament system. Thus, the model presented in this study will provide a fresh perspective on collective phenomena in intracellular transportation.

Although the detachment rule using a hard cutoff we adopted is a reasonable approximation, the exponential dependency of the detachment rate on force has been established experimentally^20^. We have also examined the exponential dependency of the detachment rate in Supplementary Information, to confirm that the results below are not altered by the choices of the exponential form.

Several experimental manipulations (e.g., increases in salt concentration or ATP/ADP concentration) correspond to the changes in parameters in our model, and the consequent results by the changes are consistent to previous experimental observations^1^. As is also discussed in the experiment^1^, increase in salt concentrations weakens the motor-microtubule interaction and hence lowers the number of microtubule-bound motors, which leads to the switch of the directionality. In our model, increases in salt concentrations is represented by the increase in the detachment rate 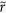, which lowers the number of microtubule-bound motors *N*_*b*_, resulting in the switch in the directionality. The directionality also depends on the length of the microtubule in the sliding assay^1^. In our model, increases in the length corresponds to the increase in the number of total motors *N*. ATP/ADP concentration affects the speed of the walk toward build-in direction, i.e., *v*_0_. It may also alters the detachment rate 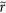, Δ_+_ and Δ_−_.

Some references have reported changes in motility of Cin8 even in a single molecule assay; Düselder *et al*.^30^ showed that if Cin8 is lacking the tail domain it loses the directionality. It has also been shown that the directionality of a single Cin8 can switch by changing the salt concentration^31^. These results suggest that switch in directionality can occur even by the structural change of a single motor under a change in external condition, without any collective effects. However, the experiment by Roostalu *et al*.^1^, in particular the experiment on a microtubule gliding assay, showed that, under fixed concentration of salt and ATP, the directionality of the full-length-Cin8 switches, depending on the concentration of motors or the length of MT. This implies that the number of motors bound to MT crucially determines the directionality. This switch in this experiment cannot be explained without cooperative effects between motors. The proposed model successfully explains the directionality switch because of a collective phenomenon even for groups of minus-end directional motors.

Furthermore, quite recently, the directionality switch of another motor, kinesin-14 KlpA, due to collective binding of MT was reported^6^. In contrast to Cin8, this motor showed plus-end directionality in a single molecule assay and minus-end directionality in MT sliding assay. Interestingly, the walk of a motor in a single molecule assay seems to display a biased random walk with a large diffusion constant, which is exactly what is expected from our theory with *v*_0_ > 0 and Δ_+_ < Δ_−_. These experimental results are also explained by our model.

In addition, an experimental study by Britto *et al*. reported the directionality switch in kinesin-5 Cut7^7^. In the case of Cut7, the directionalities in different conditions are same as Cin8: the minus-end directionality for a small number of bound motors, and the plus-end directionality for a large number of motors. As a candidate mechanism for the directionality switch, they proposed a hypothesis based on motor crowding on the microtubule lattice: they assumed that motor’s steps toward minus-end direction are selectively inhibited by collisions with interaction between neighboring motors, leading to directionality switch depending on whether motors are crowded or not. This hypothesis is fundamentally different from that proposed in the present paper, because the scenario proposed here does not need any direct interactions between motors (i.e., collisions or exclusive volume effect between neighboring motors) but requires only indirect interaction via the movement of the filament. The key difference between the consequences of the two hypotheses lies in the “density” verses “number”. In the sliding assay, the crowding hypothesis requires high “density” of kinesin so that each bound kinesin on MT can collide with the neighboring kinesins, and thus predicts that the directionality transition emerges at a relatively high concentration of kinesin and that the critical concentration of the transition is independent of the length of microtubules. In contrast, in our scenario, the directionality switch emerges depending on “number” of bound kinesin on the same MT rather than its density. Hence, the directionality can switch even for lower concentration of kinesin, and the critical concentration depends on the length of the microtubules. So far, both hypotheses can be valid, and future experiments to distinguish the above points are required.

We have demonstrated that the number of molecules, rather than the density, can dramatically alter the motility mode. These findings are related to those reported in the growing literature on the small-number effect in chemical reaction systems^32^,^33^.

Although we only considered a MT sliding assay here, the proposed mechanism is straightforwardly applicable to a situation where both ends of a motor can bind to two different MTs (i.e., a motor that can cross-link to them), as is commonly observed *in vivo*. In such a situation, the number of motors that can cross-link to the same MTs changes, depending on the configuration between the two MTs. Hence, the present mechanism provides a novel means of context-dependent regulation for a mechano-chemical coupling: plus- and minus-end directionality is used properly depending on the microtubule’s configuration. For instance, a team of cross-linkers operating under the proposed mechanism can transport two MTs in an anti-parallel alignment with their minus-end leading for a long overlap region, as abundant motors can cross-link. In contrast, for a short overlap, the motors would transport the MTs with their plus-end leading via a change in the directionality. As a result, the overlap region between two plus-ends of the MTs would be stably maintained within a certain length by only a single type of motor, which is a possible functional role of the dual-directionality of Cin8. This is in contrast to the conventional view in which two different types of motors are required for the maintenance of the overlap region, which is important for the mitotic spindle formation^28^,^34^,^35^.

## Additional Information

**How to cite this article**: Saito, N. and Kaneko, K. Embedding dual function into molecular motors through collective motion. *Sci. Rep.* **7**, 44288; doi: 10.1038/srep44288 (2017).

**Publisher's note:** Springer Nature remains neutral with regard to jurisdictional claims in published maps and institutional affiliations.

## Supplementary Material

Supplementary Information

Supplementary Video S1
