# Supplementary material for: Embedding dual function into molecular motors through collective motion
Source: Sci Rep. 2017 Mar 10;7:44288. doi: 10.1038/srep44288 (PMC5345074; doi:10.1038/srep44288)
Supplement: Supplementary Information [file srep44288-s1.pdf]

Supplementary information

for "Embedding dual function into molecular motors through collective motion"

N. Saito and K. Kaneko

1 Derivation of Eq. (3)

Here, we derive the model equation (3) in the main manuscript in a different manner. We assume that each motor bound to the filament moves via a one-dimensional random walk along the filament, the driving force of which is generated by binding of the leading head to MT associated with the motor step. The driving force is assumed to arise from thermal activation or ATP hydrolysis, and is denoted by $\eta_i = \eta(x_i, t)$, which is given in the main text. We consider the over-damped Langevin equation of the coordinate of each motor x_i and filament y as

$$\begin{aligned} \gamma_x \dot{x}_i &= -kx_i - \nu_i + \eta_i, \\ \gamma_y \dot{y} &= \sum_i^N \{\nu_i - \eta_i\} + \xi_y(t), \end{aligned} \tag{S1}$$

where γ_x and γ_y are the friction coefficients of the motor with the surrounding medium and the filament respectively, whereas k is the spring constant and $\xi_y(t)$ the thermal random force applied to the filament as $\langle \xi_y(t)\xi_y(t')\rangle = 2D_y\gamma_y^2\delta(t-t')$. ν_i represents the frictional force between the motor and the filament. It satisfies the no-slip condition, i.e., the relative distance between each bound motor and the filament does not change unless η_i causes the filament to slide. From these equations, we obtain

$$\dot{x}_i - \dot{y} = \left(\frac{1}{\gamma_x} + \frac{1}{\gamma_y}\right)\eta_i - \frac{(kx_i + \nu_i)}{\gamma_x} - \frac{1}{\gamma_y}\sum_i^N \nu_i + \frac{1}{\gamma_y}\sum_{j\neq i}^N \eta_j - \frac{\xi_y}{\gamma_y}. \tag{S2}$$

The no-slip condition is simply formulated as $\dot{x}_i - \dot{y} = (1/\gamma_x + 1/\gamma_y)\eta_i$ for each i, because $\dot{x}_i - \dot{y}$ should be zero for $\eta_i = 0$.[1] From the no-slip condition, we obtain

$$\dot{x}_i = \frac{\gamma_x + \gamma_y}{\gamma_x\gamma_y}\eta_i - \frac{k}{\gamma_y + N\gamma_x}\sum_i^N x_i - \frac{1}{\gamma_y}\frac{\gamma_y + \gamma_x}{\gamma_y + N\gamma_x}\sum_i^N \eta_i + \frac{1}{\gamma_y + N\gamma_x}\xi_y, \tag{S3}$$

$$\dot{y} = -\frac{k}{\gamma_y + N\gamma_x}\sum_i^N x_i - \frac{1}{\gamma_y}\frac{\gamma_y + \gamma_x}{\gamma_y + N\gamma_x}\sum_i^N \eta_i + \frac{1}{\gamma_y + N\gamma_x}\xi_y. \tag{S4}$$

[1] In the main text, we adopt $\dot{x}_i - \dot{y} \simeq \frac{1}{\gamma_x}\eta_i$ by assuming $\gamma_x \ll \gamma_y$.

We estimate the orders of the parameters as follows: The friction coefficient of the motor head $\gamma_x \sim 10^{-7}$ [pN·s/nm] (the friction of a sphere with the diameter ~ 10 nm in water); that of the filament $\gamma_y \sim 10^{-3}$ [pN·s/nm] (the friction of a ~ 20-μm-long microtubule in water for the sliding assay [1]); $N = 1$–10^2; and $k = 0.1$–1 [pN/nm]. In addition, η_i is estimated as $|\eta_i/\gamma_x| \sim 100$ [nm/s] from the single molecular assay of Cin8 [2]. Using these values, each term in Eq. (S3) is estimated as follows:

$$
\begin{aligned}
\left|\frac{\gamma_x+\gamma_y}{\gamma_x\gamma_y}\eta_i\right| &\sim 100 \text{ [nm/s]},\\
\left|\frac{k}{\gamma_y+N\gamma_x}\sum_i^N x_i\right| &\sim 10^2 \times 10^1 \times \sqrt{N} \text{ [nm/s]},\\
\left|\frac{1}{\gamma_y}\frac{\gamma_y+\gamma_x}{\gamma_y+N\gamma_x}\sum_i^N \eta_i\right| &\simeq N\frac{\gamma_x}{\gamma_y}\frac{\eta_i}{\gamma_x} \sim 10^{-4}\times 10^2 \times N \text{ [nm/s]},\\
\frac{1}{(\gamma_y+N\gamma_x)^2}\langle \xi_y^2(t)\rangle &\sim D_y \sim 10^4 \text{ [nm}^2/s],
\end{aligned}
\tag{S5}
$$

which allows us to neglect the third term. After applying further approximations in the form of $\gamma_y + \gamma_x \simeq \gamma_y$ and $\gamma_y + N\gamma_x \simeq \gamma_y$, we obtain Eq. (3).

2 Explicit Form of $\rho(x)$ in Eq. (5)

When $f(x)$ is given by a linear function $f(x) = a_0 x$, M_1 can be represented by $M_1 = -ax + b$. For $N = 1$, a and b are $a = a_0 + \kappa$ and $b = v_0$, whereas for infinitely large N, a and b are $a = a_0$ and $b = v_0 - \kappa\langle x\rangle$, where $\langle x\rangle = \int_{-\Delta_-}^{\Delta_+} xP(x,t)dx$. Using the imaginary error function $\text{erfi}(x) = 2\int_0^x e^{t^2}dt/\sqrt{\pi}$, the explicit form of $\rho(x)$ is given as follows:

for $x < 0$, (S6)

$$
\begin{aligned}
\rho(x) &= \frac{1}{D}\frac{\int_0^{\Delta_+}\exp\{\frac{a}{2D}(x'-\frac{b}{a})^2\}dx'}{\int_{-\Delta_-}^{\Delta_+}\exp\{\frac{a}{2D}(x'-\frac{b}{a})^2\}dx'}\int_{-\Delta_-}^{x}\exp\{\frac{a}{2D}(x'-\frac{b}{a})^2-\frac{a}{2D}(x-\frac{b}{a})^2\}dx',\\
&= \sqrt{\frac{\pi}{2Da}}e^{-\frac{a}{2D}(x-\frac{b}{a})^2}\frac{\text{erfi}[\frac{b}{\sqrt{2aD}}]+\text{erfi}[\frac{a\Delta_+-b}{\sqrt{2aD}}]}{\text{erfi}[\frac{a\Delta_+-b}{\sqrt{2aD}}]+\text{erfi}[\frac{a\Delta_-+b}{\sqrt{2aD}}]}\left(\text{erfi}[\frac{ax-b}{\sqrt{2aD}}]+\text{erfi}[\frac{a\Delta_-+b}{\sqrt{2aD}}]\right),
\end{aligned}
$$

for $x \geq 0$, (S7)

$$
\begin{aligned}
\rho(x) &= \frac{1}{D}\frac{\int_{-\Delta_-}^{0}\exp\{\frac{a}{2D}(x'-\frac{b}{a})^2\}dx'}{\int_{-\Delta_-}^{\Delta_+}\exp\{\frac{a}{2D}(x'-\frac{b}{a})^2\}dx'}\int_{x}^{\Delta_+}\exp\{\frac{a}{2D}(x'-\frac{b}{a})^2-\frac{a}{2D}(x-\frac{b}{a})^2\}dx',\\
&= \sqrt{\frac{\pi}{2Da}}e^{-\frac{a}{2D}(x-\frac{b}{a})^2}\frac{\text{erfi}[\frac{b}{\sqrt{2aD}}]-\text{erfi}[\frac{a\Delta_-+b}{\sqrt{2aD}}]}{\text{erfi}[\frac{a\Delta_+-b}{\sqrt{2aD}}]+\text{erfi}[\frac{a\Delta_-+b}{\sqrt{2aD}}]}\left(\text{erfi}[\frac{-a\Delta_++b}{\sqrt{2aD}}]-\text{erfi}[\frac{-ax+b}{\sqrt{2aD}}]\right),
\end{aligned}
$$

where $D = D_x + D_y$ for $N = 1$ and $D = D_x$ for infinitely large N. The density distribution of x is then given by $P(x) = M_0\rho$, where $M_0 = N/(\int_{-\Delta_-}^{\Delta_+}\rho(x)dx + 1/r)$.

3 Explanation of the use of $D = D_x$ in Eq.4 for infinitely large N

Through transformation of the variable according to $\tilde{x}_i = x_i - z$, where z satisfies $\dot{z} = -(N\kappa + \alpha_0)z + \xi_y/\gamma_y$, ξ_y is eliminated from the $\dot{x}_i$ equation in Eq. 3. Here, $|z| \sim \sqrt{D_y/(N\kappa + \alpha_0)}$ is negligible for large N (say ~ 100). Thus, the contribution of ξ_y can be ignored, except for the shifting of the parameters, $\Delta_\pm \to \Delta_\pm - |z|$.

4 Explanation of Video S1 in Supplementary information

Representative examples of the simulation results for $N = 1$ and 100 are depicted. The following parameters are used: $v_0 = -0.1$ [μm/s]; $D_x = 0.03$ [μm^2/s]; $D_y = 0.04$ [μm^2/s]; $\kappa = 10000$ [s^{-1}] $\Delta_+ = 0.064$ [μm]; $\Delta_- = 0.048$ [μm]; $r = 20$ [s^{-1}]; $\tilde{r} = 0$; and $a_0 = 90$ [s^{-1}].

5 Force-velocity relation for the single motor

Here, we address the force-velocity relation for the single motor under a situation in Fig. S2(a), where the motor with applied force F walks along an immobile filament. Since the filament is immobilized, the no-slip condition is written as $\dot{x}_i = \eta_i/\gamma_x = v_0 + \sqrt{2D_x}\xi_i(t) - f(x_i)$. Considering the balance with the force $F = kx$, the force-velocity relation for the single motor is obtained as

$$\langle \dot{x} \rangle = v_0 - f(F/k) = v_0 - a_0 F/k. \tag{S8}$$

The stall force is calculated as $F = v_0 k/a_0$. Figure S2(b) illustrates the force-velocity relation for the parameters in Fig.2 in the main text. Note that, in Fig. S2(b), the speed for the build-in direction of the motor (i.e., minus-end direction) is considered to be plus. The stall force in Fig. S2(b) is $F \sim 0.1$ [pN], which is much smaller than the experimentally estimated value $\sim$ 8 [pN] for known kinesins. To be compatible with the experimental estimate, reasonable choice of a_0 value will be around $a_0 \sim 2$ [s^{-1}]. Figure S2(c) illustrates the force-velocity relation for $a_0 = 2$ [s^{-1}], $k = 0.1$[pN/nm] and $v_0 = 100$ [nm/s]: in this case, the stall force is calculated as 5 [pN]. Even for such parameter, we confirmed that our conclusion is not altered as is shown in Fig. S3. In this case, the directionality switches even for $N = 2$, and the value of dy/dt does not show a monotonic decrease but overshoots at $N = 2$ (see S3(b)). This overshooting behavior of dy/dt vanishes by introducing exponential dependencies of detachment rate on force, as is described in the next section.

6 Exponential dependencies of detachment rate

Throughout the paper, we assumed the hard cutoff (i.e., Δ_+ and Δ_-) for the detachment of the motors. In contrast, the exponential dependencies of detachment rate on force ($k_{off}(F) = k_0 e^{F/F_c}$ where F_c is critical force) has been well established [3, 4]. Here we show that all results are not altered by incorporating the exponential detachment rate.

Instead of the detachment rule with the hard cutoff, we improve the spontaneous detachment rate $\tilde{r}$ as $\tilde{r}(x) = \tilde{r}_0 e^{x/\Delta_+}$ for $x \geq 0$ and $\tilde{r}_0 e^{-x/\Delta_-}$ for $x < 0$. The parameter of a_0 is set as $a_0 = 2$[s^{-1}], where the force-velocity relation illustrated in S2(c) is obtained. The results are

shown in Figs. S4, which indicate that the essentially same result as in the main text is obtained with regards to the directionality switch. In this case, the overshooting behavior of dy/dt as in S3(b) at $N = 2$ vanishes. Similar to Fig.2(b) and Fig.4(b), dy/dt monotonically decreases with N.

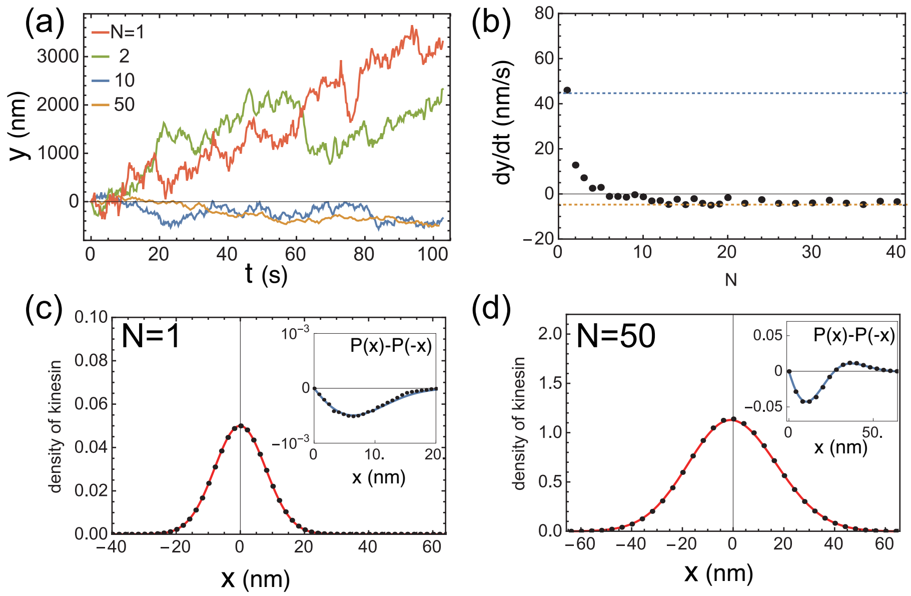

Figure S1: Behavior of the model with the asymmetric dependency of the motor's velocity upon the intramolecular strain, i.e., $|f(x)| > |f(-x)|$. (a) Time series of filament coordinate y for varying N. (b) $\dot{y}$ dependency on N. Both (a) and (b) illustrate the directionality transition with increasing N. The upper and dashed lines in (b) indicate theoretical estimates for $N = 1$ and for sufficiently large N, respectively. (c) and (d) show the x distribution $P(x)$ for $N = 1$ and 50, respectively. The lines and dots represent theoretical estimates and simulation results, respectively. The insets exhibit the difference between $P(x)$ for $x > 0$ and for $x < 0$, i.e., $P(x) - P(-x)$. The following parameters are used: $v_0 = -100$ [nm/s]; $D = 3 \times 10^4$ [nm^2/s]; $D_y = 4 \times 10^4$ [nm^2/s]; $\kappa = 1000$ [s^{-1}] ($k = 0.1$ [pN/nm] and $\gamma_y = 10^{-4}$ [pN·s/nm]); $D_+ = D_- =$ 64 [nm]; $r = 20$ [s^{-1}]; $\tilde{r} = 0$; $a_+ = 90$ [s^{-1}]; and $a_- = 106$ [s^{-1}].

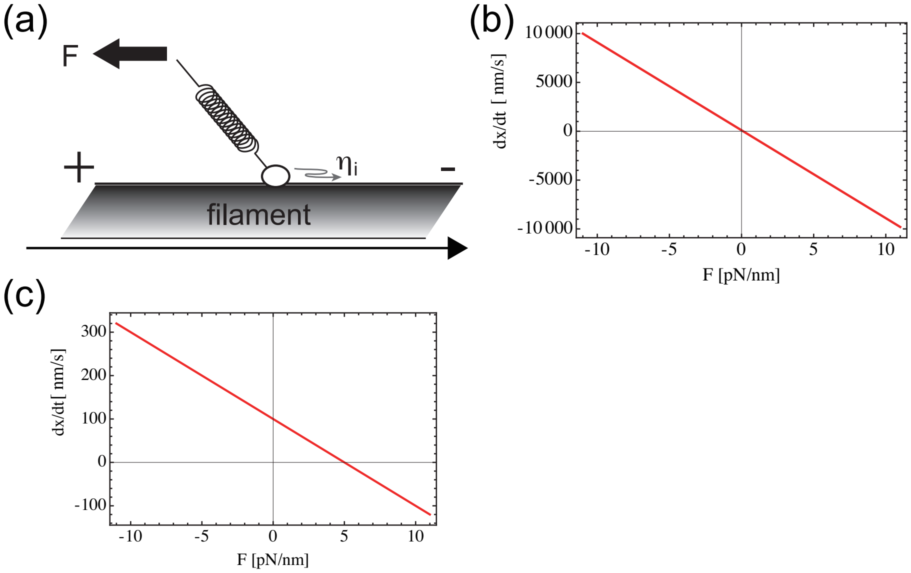

Figure S2: the force-velocity relation. (a) Schematic representation of the measurement of the force-velocity relation. (b) the force-velocity relation for $a_0 = 90$ [s^{-1}], $v_0 = 100$[nm/s] and $k = 0.1$ [pN/nm]. (c) the force-velocity relation for $a_0 = 2$ [s^{-1}], $v_0 = 100$[nm/s] and $k = 0.1$ [pN/nm].

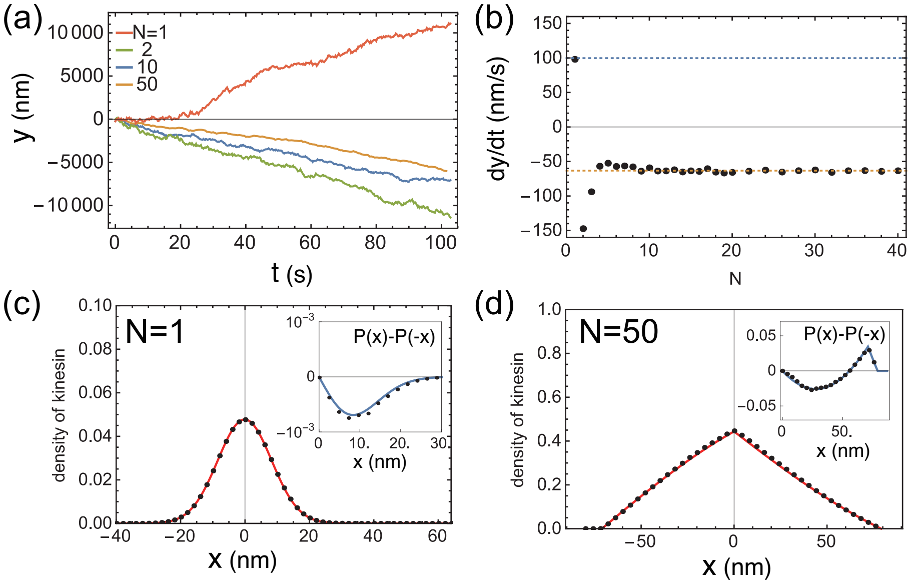

Figure S3: Behavior of the model with asymmetric strain-dependent detachment. (a) Time series of filament coordinate y for varying N. (b) $\dot{y}$ dependency on N. Both (a) and (b) exhibit directionality transitions with increasing N. The upper and bottom dashed lines in (b) indicate theoretical estimates for $N = 1$ and for a sufficiently large N, respectively. (c) and (d) show the x distribution $P(x)$ for $N = 1$ and 50, respectively. The lines and dots represent theoretical estimates and simulation results, respectively. The following parameters are used: $v_0 = -100$ [nm/s]; $D_x = 3 \times 10^4$ [nm^2/s]; $D_y = 4 \times 10^4$ [nm^2/s]; $\kappa = 1000$ [s^{-1}] ($k = 0.1$ [pN/nm] and $\gamma_y = 10^{-4}$ [pN·s/nm]); $\Delta_+ = 80$ [nm]; $\Delta_- = 72$ [nm]; $r = 20$ [s^{-1}]; $\tilde{r} = 0$; and $a_0 = 2$ [s^{-1}].

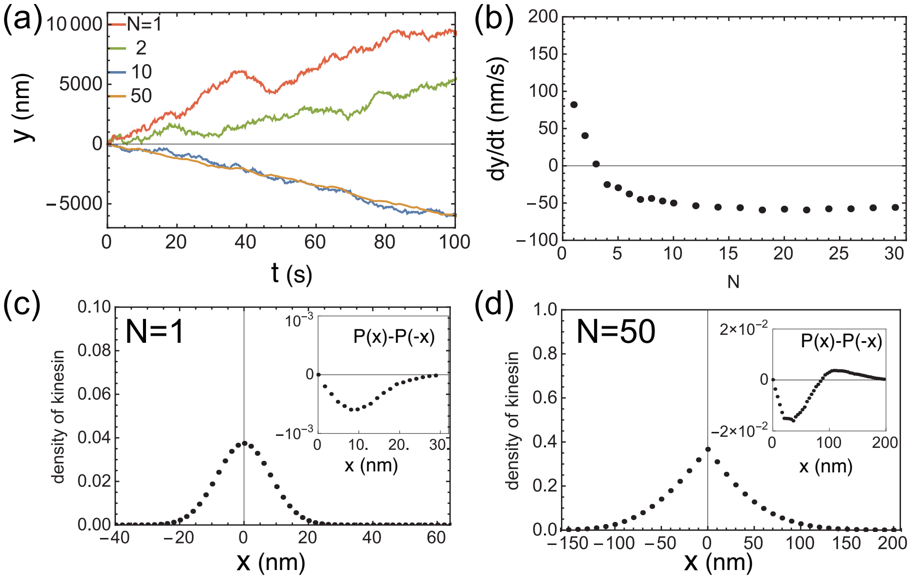

Figure S4: Behavior of model with exponential dependence of detachment rate as $\tilde{r}(x) = \tilde{r}_0 e^{x/\Delta_+}$ for $x \geq 0$ and $\tilde{r}_0 e^{-x/\Delta_-}$ for $x < 0$. (a) Time series of filament coordinate y for varying N. (b) $\dot{y}$ dependency on N. Both (a) and (b) exhibit directionality transitions with increasing N. (c) and (d) show the x distribution $P(x)$ for $N = 1$ and 50, respectively. The dots represent simulation results. The following parameters are used: $v_0 = -100$ [nm/s]; $D_x = 3 \times 10^4$ [nm^2/s]; $D_y = 4 \times 10^4$ [nm^2/s]; $\kappa = 1000$ [s^{-1}] ($k = 0.1$ [pN/nm] and $\gamma_y = 10^{-4}$ [pN·s/nm]); $\Delta_+ = 80$ [nm]; $\Delta_- = 48$ [nm]; $r = 20$ [s^{-1}]; $\tilde{r}_0 = 5$ [s^{-1}]; and $a_0 = 2$ [s^{-1}].

References

[1] Jonathon Howard et al. *Mechanics of motor proteins and the cytoskeleton.* Sinauer Associates Sunderland, MA, 2001.

[2] Johanna Roostalu, Christian Hentrich, Peter Bieling, Ivo A Telley, Elmar Schiebel, and Thomas Surrey. Directional switching of the kinesin cin8 through motor coupling. *Science*, 332(6025):94–99, 2011.

[3] Nick J Carter and RA Cross. Mechanics of the kinesin step. *Nature*, 435(7040):308–312, 2005.

[4] Johan OL Andreasson, Bojan Milic, Geng-Yuan Chen, Nicholas R Guydosh, William O Hancock, and Steven M Block. Examining kinesin processivity within a general gating framework. *Elife*, 4:e07403, 2015.
