# Supplementary figures and images for: Embedding dual function into molecular motors through collective motion
Source: Sci Rep. 2017 Mar 10;7:44288. doi: 10.1038/srep44288 (PMC5345074; doi:10.1038/srep44288)

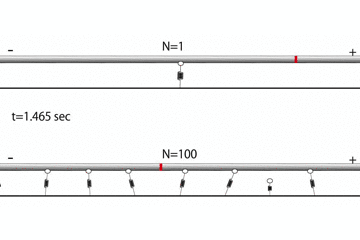

Supplement: Supplementary Video S1 [file srep44288-s2.gif]
